# In Situ Exfoliation and Pt Deposition of Antimonene for Formic Acid Oxidation via a Predominant Dehydrogenation Pathway

**DOI:** 10.34133/2020/5487237

**Published:** 2020-02-21

**Authors:** Yiqiong Zhang, Man Qiao, Yucheng Huang, Yuqin Zou, Zhijuan Liu, Li Tao, Yafei Li, Chung-Li Dong, Shuangyin Wang

**Affiliations:** ^1^State Key Laboratory of Chemo/Bio-Sensing and Chemometrics, College of Chemistry and Chemical Engineering, Hunan University, Changsha 410082, China; ^2^Jiangsu Collaborative Innovation Centre of Biomedical Functional Materials, School of Chemistry and Materials Science, Nanjing Normal University, Nanjing, China; ^3^Department of Physics, Tamkang University, Tamsui 25137, Taiwan

## Abstract

Direct formic acid fuel cell (DFAFC) has been considered as a promising energy conversion device for stationary and mobile applications. Advanced platinum (Pt) electrocatalysts for formic acid oxidation reaction (FAOR) are critical for DFAFC. However, the oxidation of formic acid on Pt catalysts often occurs via a dual pathway mechanism, which hinders the catalytic activity owing to the CO poisoning. Herein, we directly exfoliate bulk antimony to 2D antimonene (Sb) and *in situ* load Pt nanoparticles onto antimonene sheets with the assistance of ethylenediamine. According to the Bader charge analysis, the charge transfer from antimonene to Pt occurs, confirming the electronic interaction between Pt and Sb. Interestingly, antimonene, as a cocatalyst, alters the oxidation pathway for FAOR over Pt catalyst and makes FAOR follow the more efficient dehydrogenation pathway. The density functional theory (DFT) calculation demonstrates that antimonene can activate Pt to be a lower oxidative state and facilitate the oxidation of HCOOH into CO_2_ via a direct pathway, resulting in a weakened intermediate binding strength and better CO tolerance for FAOR. The specific activity of FAOR on Pt/Sb is 4.5 times, and the mass activity is 2.6 times higher than the conventional Pt/C.

## 1. Introduction

Direct formic acid fuel cells (DFAFCs) have been considered as ideal electrochemical energy conversion devices [1, 2]. Pt-based catalysts have been widely used for formic acid oxidation reaction (FAOR) [3]. However, the oxidation of formic acid on Pt catalysts often occurs through a dual pathway mechanism (*i.e.*, dehydrogenation and dehydration pathway), which hinders the catalytic activity because of the poisoning of CO intermediates from the dehydration pathway [4, 5]. Recently, great efforts have been made to alleviate CO poisoning [6–10]. Lin et al. synthesized a Pt/Au bimetallic nanocrystal for FAOR, exhibiting good CO tolerance and high catalytic performance through the direct dehydrogenation pathway [11, 12]. Recently, antimonene as an emerging 2D-layered material has attracted great attention for electrochemical applications [13, 14], owing to its high surface area, good conductivity, large interlayer channel size, and thermodynamic stability [15]. For example, the shear-exfoliated Sb nanosheets exhibited the enhanced catalytic performance for electrochemical oxygen reaction and hydrogen evolution reaction [16]. Few layer antimonene possessed high charge-storing abilities and good cycling capabilities for supercapacitor applications [17]. For antimonene with a metallic-layered structure, it would be expected to serve as suitable supporting materials of Pt nanoparticles for FAOR. The unique electronic properties of antimonene may result in unexpected interaction with Pt and lead to promising catalytic performance.

Herein, we directly exfoliate bulk antimony to 2D antimonene nanosheets and *in situ* load Pt nanoparticles onto antimonene sheets with the assistance of ethylenediamine. In the composite, the 2D antimonene acts as the Pt support and cocatalyst for FAOR over Pt. The exfoliated Sb nanosheets have an ultrathin structure and can well anchor the *in situ* deposited Pt nanoparticles. According to the Bader charge analysis, there is a 0.24 |*e*| charge transfer from antimonene to Pt, confirming the electronic interaction between Pt and Sb. Moreover, the Sb cocatalyst can alter the oxidation pathway of formic acid over Pt catalyst and make FAOR prefer the more efficient dehydrogenation pathway. The DFT results demonstrated that the antimonene can effectively activate Pt to be a lower oxidative state and facilitate the electrooxidation of HCOOH into CO_2_*via* direct mechanisms, resulting in a weakened intermediate binding strength and the better CO tolerance for FAOR. As a result, the specific activity of FAOR on Pt/Sb is 4.5 times and the mass activity is 2.6 times higher than Pt/C.

## 2. Results and Discussion

**Figure 1:**
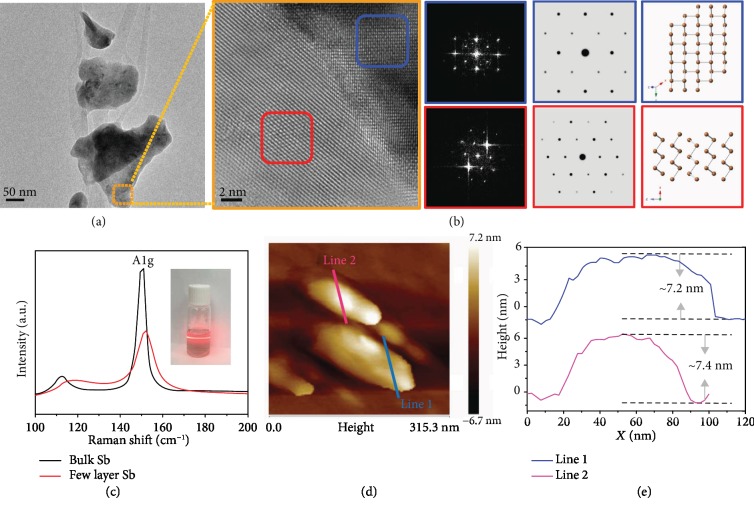
Characterization of antimonene sheets. (a) TEM image of few-layer antimonene sheets. (b) HR-TEM image of few-layer antimonene (left), the FFT patterns corresponding to the two main regions (middle) and their corresponding simulated electron diffraction patterns and crystal models (right). (c) Raman spectra of bulk Sb and few-layer Sb nanosheets. Inset: photograph of a dispersion of exfoliated antimonene showing the Faraday-Tyndall effect. (d) The AFM image and (e) the thickness of few-layer antimonene.

The bulk Sb was firstly treated by ball milling to obtain microcrystals ([Supplementary-material supplementary-material-1]). The antimonene sheets could be exfoliated by the solvothermal reaction with ethylenediamine (EDA) as intercalating agents to enlarge the interlayer spacing [18]. As shown in Figure 1(a), the antimonene has a typical nanosheet structure. HRTEM and the corresponding fast Fourier transform (FFT) images are given in Figure 1(b) to show the crystalline feature of antimonene. The FFT images of the two main regions well matched the simulated FFT pattern. As shown by the Raman spectra in Figure 1(c), there are two main peaks about Eg and A1g mode of few-layer Sb similar to that of bulk Sb. The peak intensities become weaker after exfoliation, indicating the decrease in thickness of exfoliated Sb nanosheets. Both Eg and A1g peak of the exfoliated Sb shift to the higher wavenumber region, indicating the decrease of the thickness of Sb. The exfoliation of Sb under solvothermal reaction was also confirmed by atomic force microscopy (AFM). As shown in Figures 1(d), 1(e), and [Supplementary-material supplementary-material-1], the thicknesses of antimonene is about 7 nm, which confirms the effective exfoliation of Sb nanosheets.

**Figure 2:**
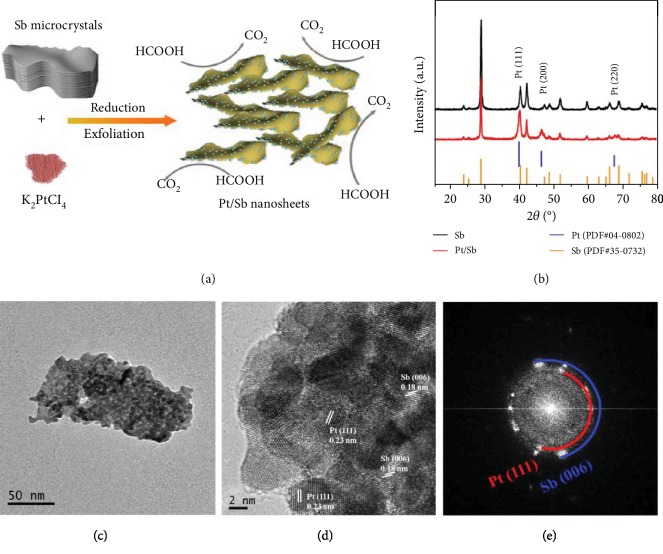
Characterization of Pt/Sb. (a) The formation process of Pt/Sb. (b) XRD spectra of Pt/Sb and Sb. (c) TEM images of Pt/Sb. (d) HRTEM image of Pt/Sb. (e) The FFT patterns of Pt/Sb.

To realize the *in situ* exfoliation of antimonene and Pt deposition on antimonene nanosheets, Pt salts were directly introduced into the exfoliation solution of Sb microcrystals and EDA. EDA has been reported to serve as the reducing agent. In the presence of Pt ions in the exfoliation solution, when few-layer antimonene sheets are exfoliated from Sb microcrystals by EDA, the Pt ions will be *in situ* reduced by EDA and Pt nanoparticles will be deposited on the surface of antimonene nanosheets (Figure 2(a)). Powder X-ray diffraction (XRD) was used to characterize the crystalline phase of Sb and Pt/Sb nanosheets. As shown in Figure 2(b), the XRD pattern of Sb and Pt/Sb nanosheets exhibits the same distinct diffraction peaks, which are well indexed to the standard card of the Sb crystal (PDF#35-0732), while the diffraction peaks at 39.7°, 46.2°, and 67.4° can be indexed to the (111), (200), and (220) planes of the Pt crystal (PDF#04-0802) for Pt/Sb, indicating the successful in situ deposition of Pt on the antimonene sheets. TEM images confirmed the structure and composition of the Pt/Sb nanosheets. As shown in Figures 2(c) and [Supplementary-material supplementary-material-1], the Pt nanoparticles are uniformly dispersed on antimonene sheets. The HRTEM and FFT images show the lattice spacing of 0.18 nm and 0.23 nm corresponding to the Sb (006) and Pt (111) planes, respectively (Figures 2(d) and 2(e)). The STEM elemental mapping analysis confirms the uniform distribution of Pt nanoparticles on the Sb nanosheets ([Supplementary-material supplementary-material-1]). The EDX result reveals the Pt/Sb ratio is around 23.88/76.11 ([Supplementary-material supplementary-material-1]), consistent with the result from inductively coupled plasma-atomic emission spectrometry (ICP-AES). For comparison, the conventional Pt/C catalysts were also prepared ([Supplementary-material supplementary-material-1]). The chemical states of the Pt nanoparticles are determined from the Pt 4f XPS spectrum which is fitted with the spin-orbit split 4f_7/2_ and 4f_5/2_ components ([Supplementary-material supplementary-material-1]). Notably, the binding energy of Pt 4f for the Pt/Sb has a slightly negative shift compared to that of the Pt/C as a result of local charge density change due to the charge transfer interaction with antimonene.

**Figure 3:**
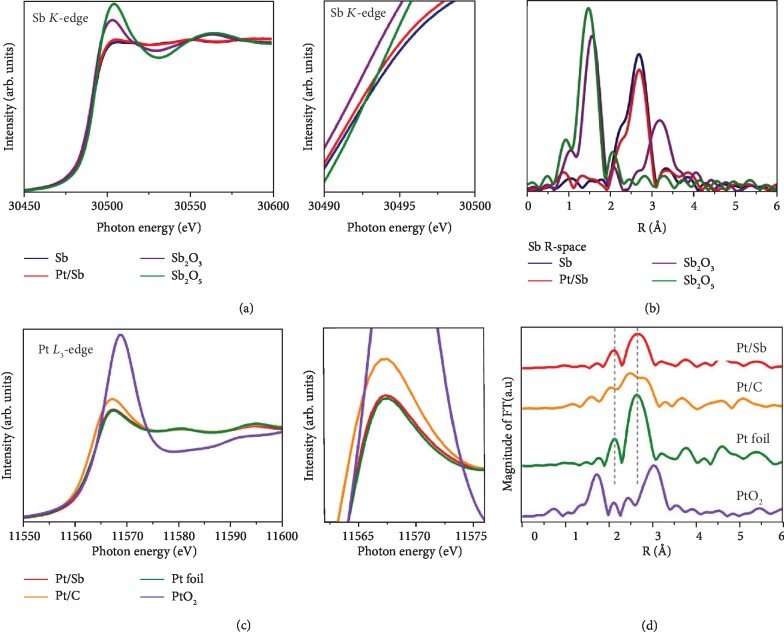
X-ray absorption spectrometric studies of Pt/Sb. (a) XANES spectra at the Sb *K*-edge of Sb, Pt/Sb, Sb_2_O_3_, and Sb_2_O_5_. (b) Fourier transform EXAFS spectrum of the Pt/Sb in comparison with Sb, Sb_2_O_3_, and Sb_2_O_5_ at Sb *R*-space. (c) XANES spectra at the Pt *L*-edge of the Pt/Sb, Pt/C, Pt foil, and PtO_2_. (d) Fourier transform EXAFS spectrum of the Pt/Sb in comparison with Pt/C, Pt foil, and PtO_2_ at Pt *R*-space.

To probe the local environment of Pt and antimonene, X-ray absorption spectroscopy (XAS) measurements were performed at the Sb *K*-edge and Pt *L*-edge. The X-ray absorption near edge structure (XANES) region of the XAS spectrum provides information about the chemical state of Sb and Pt. The Sb *K*-edge absorption edge position for Pt/Sb was similar to that of the Sb sample (Figure 3(a)), which was at higher photon energies than Sb_2_O_3_, indicating that the Sb persists its crystal structure either with Pt deposition or with the exfoliation process, but the peak at about 30504 eV shows an obvious increase in intensity for Pt/Sb compared with Sb, which reflects the strong Pt-Sb interaction with the dynamic varieties of the electronic state. Notably, the main enhanced absorption peak in Pt/Sb is higher than in Sb, which indicates that the Sb site may lose some charges, shifting the absorption peak as well as the absorption edge to higher energy. Therefore, variations in dipole strength prohibit small features from being simply attributed to the 5p unoccupied state, but to the degree of symmetry of the coordinated environment. Further structural information was obtained from Sb *K*-edge and Pt *L*-edge extended X-ray absorption fine structure (EXAFS) analyses. Fourier transformed *R*-space curves of the Sb *K*-edge EXAFS spectra clearly revealed the bonding environment of Sb atom in antimonene (Figure 3(b)). The Sb sample showed an intense Sb-Sb feature at around 2.75 Å, which was present at the *R*-space spectrum of Pt/Sb, confirming major Sb-Sb bonds (i.e., Sb nanosheets) were present in the antimonene which agreed well with the HRTEM data for Pt/Sb. The Pt *L*-edge absorption edge position for Pt/Sb was similar to that of Pt foil metal (Figure 3(c)), which was at lower photon energies than PtO_2_. Moreover, the Pt *L*-edge for Pt/C is more inclined to the state of PtO_2_. This inclined intensity is owing to the charge redistribution from Pt to C since C has higher electronegativity than Pt does. On the other hand, the first shell peak for PtO_2_ was Pt-O coordination at about 1.8 Å (Figure 3(d)), while the first shell peaks for Pt foil are around 2.2 Å and 2.65 due to Pt-Pt coordination. The EXAFS analysis based on a Pt-Pt structure was carried out to further confirm the coordination environment of the Pt metal atoms. This result indicates that the local atomic environments of Pt in Pt/Sb are resemble to Pt foil but exhibit lower coordination numbers since the intensity of the Fourier-transformed amplitude declines. The Fourier transform *k*^3^-weighted extended X-ray absorption fine structure (EXAFS) spectrum of the Pt/Sb (Pt/C) shows that the first peak is attributable to Pt-Sb (Pt-C) coordination and the second peak is attributable to Pt-Pt (Pt-Pt) coordination. The quantitative curve fittings were carried out in the *k*^3^-weighted EXAFS oscillation. [Supplementary-material supplementary-material-1] shows the *k*^3^-weighted EXAFS spectra from *k* = 2.5 to 13.5 Å^−1^ of Pt/Sb (A) and Pt/C (B) (black solid lines). The best fits (red dash lines) to the spectra are also displayed in [Supplementary-material supplementary-material-1]. The results of the best fits are given in [Supplementary-material supplementary-material-1]. The Pt-Sb bond distance, according to the best fits to the *k*^3^-weighted EXAFS spectra of Pt/Sb is 2.12 Å. Notably, the differences between the Pt-Pt distances in Pt/Sb and Pt/C are evidenced. The Pt-Pt bond distance in Pt/Sb is larger than that in Pt/C.

**Figure 4:**
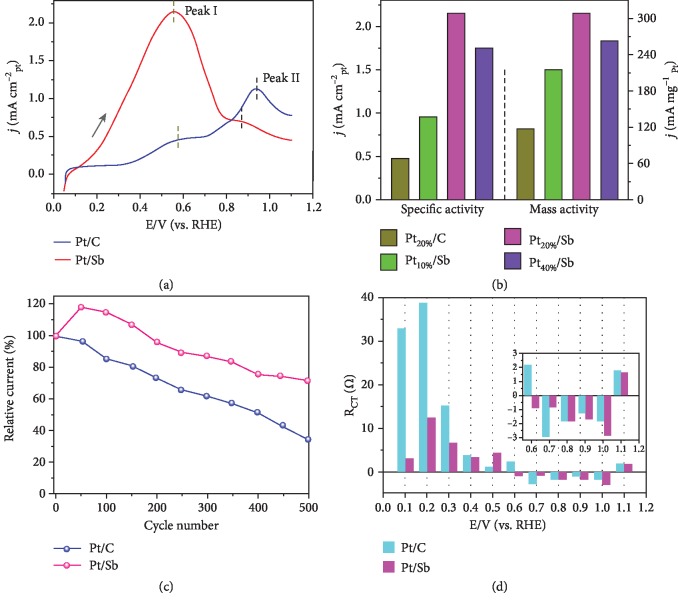
Electrochemical performance. (a) Normalized cyclic voltammograms of FAOR on Pt/Sb and Pt/C in 0.1 M HClO_4_+0.1 M HCOOH at 50 mV s^−1^. (b) The specific activity and mass activity of catalysts. (c) The stability of Pt/Sb and Pt/C. (d) Charge-transfer resistance of FAOR at different potentials on Pt/C and Pt/Sb.

To study the formic acid oxidation reaction mechanism on Pt/Sb, the as-prepared Pt/Sb and Pt/C electrocatalysts are tested for formic acid oxidation. [Supplementary-material supplementary-material-1] shows the cyclic voltammogram (CV) curves for Pt/Sb and Pt/C in a N_2_-saturated 0.1 M HClO_4_ at a scan rate of 50 mV s^−1^. The electrocatalytic properties of Pt/Sb and Pt/C toward FAOR are investigated in a N_2_-saturated 0.1 M HClO_4_ and 0.1 M HCOOH. As shown in Figure 4(a), the onset potential of Pt/Sb is much lower than that of Pt/C and the oxidation current of formic acid on Pt/Sb is much higher than that of Pt/C, indicating that Pt on antimonene sheets can significantly improve the electrocatalytic activity for formic acid oxidation. For Pt/Sb, the forward scan of the CV curves for the formic acid oxidation is characterized by a strong current peak at ~0.55 V and a shoulder at ~0.87 V. Similar to the reaction on Pt-based catalysts, the first peak can be assigned to the direct oxidation of formic acid to form CO_2_, while the second peak is related to the oxidation of the intermediate CO generated from the dissociative adsorption step. For Pt/C, the peak I at 0.60 V and peak II at 0.96 V are corresponding to the oxidation of formic acid *via* the dehydrogenation pathway and oxidation of CO_ads_ formed *via* the dehydration pathway, respectively. In addition, the ratio *R* between peak I and peak II is often used to determine the pathway of FAOR [19]. For Pt/C, peak I current is 2.75 times lower than peak II current (*R* = 0.36), indicating that the dehydration pathway is predominant. For Pt/Sb, peak I current is 3.07 times higher than peak II current (*R* = 3.07), illustrating that the FAOR on Pt/Sb accomplishes mainly through the dehydrogenation pathway. As shown in [Supplementary-material supplementary-material-1], the Pt/Sb catalyst apparently shows larger ratio of I/II than that of other reported catalysts. These results indicate that the indirect pathway is dehydration reaction to form CO poisoning intermediates which is mostly suppressed on the Pt/Sb and the majority of formic acid is directly oxidized via a dehydrogenation pathway. Similar results are also obtained for different Pt ratio on the Sb nanosheets ([Supplementary-material supplementary-material-1]).

The comparison of the specific activity and mass activity of Pt/Sb and Pt/C catalysts is shown in Figure 4(b). The specific activity of FAOR for Pt_20%_/Sb is about 2.15 mA cm^−2^, which is much higher than those for Pt_20%_/C (0.47 mA cm^−2^), Pt_40%_/Sb (1.74 mA cm^−2^), and Pt_10%_/Sb (0.95 mA cm^−2^). The mass activity (the current density is normalized by the Pt mass) for Pt_20%_/Sb is about 308.6 mA mg^−1^, which is much higher than those for Pt_20%_/C (116.6 mA mg^−1^), Pt_40%_/Sb (262.2 mA mg^−1^), and Pt_10%_/Sb (215.4 mA mg^−1^), confirming that Pt_20%_/Sb possesses higher specific and mass activity toward the FAOR. Moreover, the electrocatalytic activity of the Pt/Sb samples toward CO oxidation was compared with that of the Pt/C catalyst. As shown in [Supplementary-material supplementary-material-1], both the onset potential and peak potential for the oxidation of CO_ads_ on Pt/Sb samples are shifted negatively, suggesting that the CO_ads_ is more easily oxidized on Pt/Sb when compared with that of Pt/C. Therefore, the electronic interaction between Pt and Sb can facilitate the oxidation of the CO poisoning intermediates, resulting in significantly improved electrocatalytic activity for the FAOR. Chronoamperometry curves of Pt/Sb and Pt/C are also measured to evaluate the rate of surface poisoning. [Supplementary-material supplementary-material-1] shows that the Pt/Sb catalysts exhibit a slower current decay over time in comparison with the Pt/C catalysts and the current density of Pt_20%_/Sb is higher than that of Pt_40%_/Sb, Pt_10%_/Sb, and Pt/C catalysts through the entire range. After 500 cycles, the peak current of Pt/Sb remains 71.9% of its initial value, while Pt/C only remains 34.8%, further revealing the enhanced stability performance for FAOR (Figure 4(c)). After the stability testing, the morphologies of Pt/Sb are retained well, indicating the high structural stability of Pt nanoparticles on antimonene sheets ([Supplementary-material supplementary-material-1]).

Electrochemical impedance spectroscopy (EIS) was used to investigate the kinetics of FAOR at different potentials. The impedance data in different potential range are fitted in Figures [Supplementary-material supplementary-material-1] and [Supplementary-material supplementary-material-1], and the fitting results are shown in Tables [Supplementary-material supplementary-material-1] and [Supplementary-material supplementary-material-1]. Figures [Supplementary-material supplementary-material-1] and [Supplementary-material supplementary-material-1] show the Nyquist plots of the Pt/C and Pt/Sb in 0.1 M HCOOH+0.1 M HClO_4_ with varied electrode potentials, respectively. On Pt/C, the impedance arcs are located within the first quadrant, and the diameter of the arcs decreases with increasing potential from 0.2 to 0.5 V, suggesting the faster electron transfer rate of FAOR at higher potential. In accordance with the impedance, the oxidation current density increases with increasing potential as shown in Figure 4(a). However, with the potential further increase, the diameter of impedance arcs increases first and then negative impedance was observed in the second quadrant. Such negative impedance has been found previously for FAOR on Pt-based electrocatalysts [20–22] and was ascribed to the formation of chemisorbed hydroxyl species at electrode surface, which is attributed to the (pseudo-) inductive characteristics upon the oxidative removal of the adsorbed CO intermediate, consistent with the voltammetric response where a small anodic shoulder is observed in the potential range. At more positive electrode potentials (1.1 V), the impedance arcs return to the first quadrant. Similar impedance features were observed with Pt/Sb, although the appearance of negative impedance started to occur at somewhat different potentials, as reflected by the variation of the charge transfer resistance (*R*_CT_) with electrode potentials. Figure 4(d) depicts the variation of *R*_CT_ with electrode potentials at Pt/Sb and Pt/C. It can be seen that the appearance of (pseudo-) inductive characters (i.e., negative *R*_CT_) coincided with the oxidation of adsorbed poisonous CO. [23] Therefore, the onset potentials of negative *R*_CT_ might be exploited to compare the tolerance to CO poisoning of Pt/Sb and Pt/C. In Figure 4(d), the onset potential of negative *R*_CT_ in Pt/Sb is lower than that of Pt/C, which indicates that Pt/Sb represents the better CO tolerance compared to Pt/C. In addition, we also performed EIS at the open circuit potential (before electrochemical cycling) in 0.1 M HClO_4_ ([Supplementary-material supplementary-material-1]). The charge transfer resistance of Pt/Sb is lower than that of Pt/C, indicating better charge transport for Pt/Sb.

**Figure 5:**
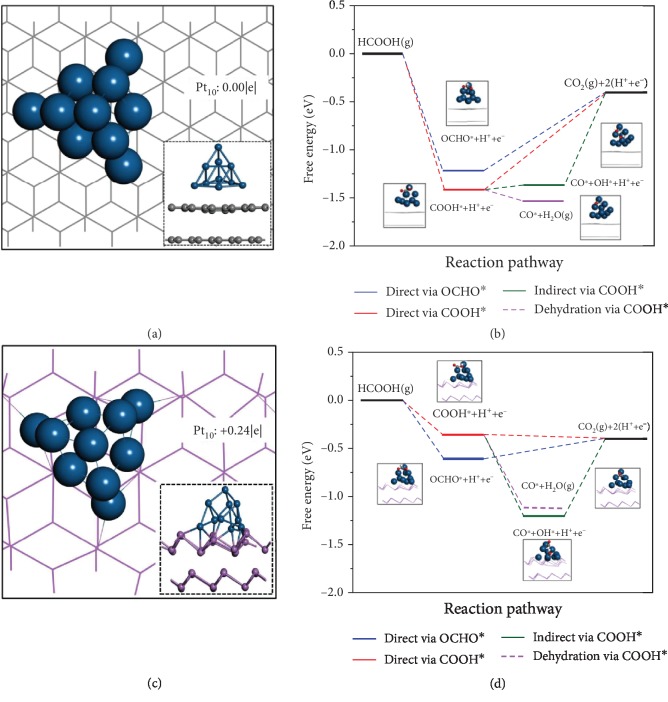
The density functional theory (DFT) calculations. Top and side views of optimized Pt_10_ cluster on (a) graphene and (c) Sb support. The charge transfers are labeled by red words. Blue, purple, and gray balls present Pt, Sb, and C atoms, respectively. Gray and purple lines denote the graphene and antimonene, respectively. The calculated free energy diagrams for FAOR on (b) Pt/C and (d) Pt/Sb. Blue, white, gray, and red balls present Pt, H, C, and O atoms, respectively. Gray and purple lines denote graphene and antimonene, respectively.

To identify the origin of the high FAOR activity of Pt/Sb, density functional theory (DFT) calculations were performed to unveil the interaction between Pt and Sb and its impact on the catalytic performance. As shown in Figures 5(a) and 5(c), Pt_10_ cluster can bind strongly with the first atomic layer of Sb sheet. According to the Bader charge analysis, there is a 0.24 |*e*| charge transfer from the Sb sheet to Pt_10_ cluster. In sharp contrast, there is no evident charge transfer that occurs in Pt/C, confirming the electronic interaction between Pt and Sb. To elucidate the different catalytic performance of negatively charged Pt^*δ*-^ on Pt/Sb, neutral Pt^0^ on Pt/C, and pure Pt (111), we calculated the reaction free energies for electrochemical steps involved in the FAOR process (Figures 5(b), 5(d), and [Supplementary-material supplementary-material-1]). Generally, FAOR follows the dual path mechanism, in which HCOOH can be oxidized directly to CO_2_*via* a reactive species (COOH^∗^ or OCHO^∗^), or indirectly *via* forming an adsorbed CO^∗^ species. We considered the indirect pathway via the COOH^∗^ instead of OCHO^∗^ intermediates as the former is energetically more favorable for the formation of CO^∗^ [24, 25]. Therefore, there are three possible paths for CO_2_ formation, including direct OCHO^∗^ mechanism (path1), direct COOH^∗^ mechanism (path2), and indirect COOH^∗^ dehydration mechanism (path3).  Figure 5(b) shows the free energy diagram of Pt/C. Both OCHO^∗^ and COOH^∗^ intermediates bind with surface strongly. As a result, the formation of CO_2_ formation is a rate-determining step (RDS) for the direct mechanism. The theoretically determined onset potential for the OCHO^∗^ and COOH^∗^ mechanism is 0.81 and 1.01 V, respectively. Moreover, the RDS of indirect mechanism on Pt/C is the conversion of CO^∗^ and OH^∗^ to CO_2_ with an onset potential of 0.97 V. Therefore, the higher potentials for three mechanisms on Pt/C indicate its poor activity and selectivity toward FAOR. We next investigated the FAOR on Pt/Sb. As shown in Figures 5(d) and [Supplementary-material supplementary-material-1], Pt/Sb can trigger the spontaneous deprotonation of COOH^∗^ to CO_2_ or decomposition of OCHO^∗^ to CO_2_ with an onset potential of 0.21 V. However, the onset potential of the indirect mechanism on Pt/Sb is as high as 0.81 V. Therefore, the lower onset potential of COOH^∗^ and OCHO^∗^ pathways will facilitate the electrooxidation of HCOOH into CO_2_ via direct mechanisms. Remarkably, compared to Pt/C, the CO^∗^ poisoning is much less pronounced in Pt/Sb due to the weaker CO^∗^ adsorption (-1.13 eV for Pt/Sb and -1.54 eV for Pt/C). Overall, our computations vividly demonstrated Pt on antimonene can effectively activate Pt to be lower oxidative states, resulting in a weakened intermediate binding and the enhanced FAOR performance.

## 3. Conclusions

In summary, we have realized the *in situ* exfoliation and Pt deposition of antimonene as advanced electrocatalysts for the formic acid oxidation. We directly exfoliated Sb nanosheets and deposited the *in situ* reduced Pt nanoparticles onto the exfoliated Sb nanosheets by EDA. Theoretical analysis demonstrated the charge transfer from Sb to Pt, confirming the electronic interaction between Pt and Sb. The Pt/Sb catalyst achieved remarkable catalytic performance for FAOR compared with the Pt/C. The Sb cocatalysts can alter the oxidation pathway of formic acid over Pt catalyst and prefer the dehydrogenation pathway reaction. Moreover, the computations demonstrated that Pt on antimonene can effectively activate Pt to be lower oxidative states and facilitate the electrooxidation of HCOOH into CO_2_ via direct mechanisms, resulting in a weakened intermediate binding strength and the better CO tolerance of Pt/Sb. As a result, the specific activity for Pt/Sb is about 4.5 times and the mass activity is about 2.6 times higher than those for Pt/C. This work would provide valuable ideas to design and prepare advanced Pt-based electrocatalysts for direct formic acid fuel cells.

## 4. Materials and Methods

### 4.1. Materials and Chemicals

All the chemicals were used as purchased without further purification. Commercially available antimony material (99.9999%, metal basis), ethylenediamine (EDA, >99%), benzene, and ethanol were purchased from Shanghai Chemical Reagent Co. Ltd. Potassium tetrachloroplatinate (K_2_PtCl_4_) was purchased from Shanghai Aladdin Biochemical Technology Co., Ltd. and Nafion (5 wt %) from Sigma-Aldrich. Doubly distilled deionized water (DIW, 18.2 M*Ω*) was used for all preparations.

### 4.2. Preparation of Few Layer Antimonene

Firstly, the bulk antimony was submitted to a ball milling reactor matched with quality stainless steel balls under the filling of Ar (with a purity of 99.999%) atmosphere and treated for 180 min to obtain the Sb microcrystals. Then, the few layer antimonene sheets were synthesized by a direct solvothermal process employing the Sb microcrystals as raw materials and ethylenediamine as solvent. In a typical synthesis process, 30 mg Sb powders were dissolved in ethylenediamine and sonicated for 120 min in an ice bath and further transferred to a 50 mL Teflon liner, followed by heating at 140°C for 12 h. After cooling to room temperature naturally, the product was collected and washed with benzene, ethanol, and distilled water in sequence, followed by drying in a vacuum at 60°C overnight.

### 4.3. Preparation of Pt/Sb Nanosheets

Similarly, the Pt/Sb were synthesized by a direct solvothermal process employing the resulting Sb microcrystals and K_2_PtCl_4_ as raw materials and ethylenediamine as solvent. In a typical synthesis process, 30 mg Sb microcrystals were dissolved in ethylenediamine and sonicated for 120 min in an ice bath. Then, the appropriate amount of K_2_PtCl_4_ (20 mg mL^−1^) was added into the solution under vigorous stirring for 30 min and further transferred to a 50 mL Teflon liner, followed by heating at 140°C for 12 h. After cooling to room temperature naturally, the product was collected and washed with benzene, ethanol, and distilled water in sequence, followed by drying in a vacuum at 60°C overnight. For the synthesis of Pt/C, the similar procedure was followed with replacing the Sb to carbon powders (Vulcan XC-72). The Pt loading is 10.8% for Pt_10%_/Sb, 22.1% for Pt_20%_/Sb, 44.3% for Pt_40%_/Sb, and 21.4% for Pt_20%_/C analyzed by ICP, respectively.

### 4.4. Material Characterization

X-ray powder diffraction (XRD) characterization was carried out on a Siemens D500 diffractometer with a Cu K*α* radiation. The morphology and microstructure of all samples were investigated transmission electron microscopy (TEM, FEI, F20, S-TWIX) and scanning electron microscope (SEM, Hitachi, S-4800). The actual loading of Pt in the catalysts was determined by inductively coupled plasma optical emission spectroscopy (ICP-OES, Spectro Blue Sop, German). The size and thickness of electrocatalysts were determined by an atomic force microscope (AFM, Bruker Bioscope System). The Raman spectra were recorded at room temperature on a Horiba HR 800 with an argon ion laser operating at 532 nm. X-ray photoelectron spectroscopy (XPS) measurements and analysis were recorded on a Thermo Fisher-VG Scientific (ESCALAB 250Xi) photoelectron spectrometer. X-ray absorption spectra at Sb *K*-edge EXAFS were, respectively, recorded at the Taiwan Light Source (TLS) beamlines 01C1 of the National Synchrotron Radiation Research Center, Hsinchu, Taiwan. EXAFS data were collected in a fluorescent mode at room temperature.

### 4.5. Electrochemical Measurements

Electrochemical measurements of Pt/Sb and Pt/C for formic acid oxidation reaction were conducted at room temperature in a standard three-electrode cell on a CHI760e electrochemical workstation. A glassy carbon electrode (GCE, 6 mm in diameter) was used as the working electrode; a carbon electrode and saturated calomel electrode were used as the counter and reference electrode, respectively. 4 mg of catalyst (Pt_10%_/Sb, Pt_20%_/Sb, Pt_40%_/Sb, and Pt_20%_/C) was dispersed in 1 mL of isopropanol and ultrasonicated for 60 min. Then, 50 *μ*L of 5 wt% Nafion solution was added in the slurry followed by ultrasonication for another 60 min. After that, the GCE working electrode was coated with the above catalyst ink and dried naturally. The cyclic voltammograms (CVs) were obtained in nitrogen-saturated 0.1 M HClO_4_ at the scan rate of 50 mV s^−1^ in a potential window of -0.25-0.8 V versus SCE. The elelctrocatalytic activity for the formic acid oxidation reaction was measured in a nitrogen-saturated 0.1 HClO_4_ and 0.1 M HCOOH solution at a scan rate of 50 mV s^−1^. The solution was purged with ultra-high-purity N_2_ before the electrochemical testing. The electrocatalytic activity of all Pt/Sb catalysts toward CO oxidation was contrasted with the Pt/C catalyst in 0.1 M HClO_4_ electrolyte at a scan rate of 10 mV s^−1^. High purity CO was bubbled into the electrolyte solution for 20 min while keeping the electrode potential at -0.1 V versus SCE to achieve maximum coverage of CO at the Pt centers. Dissolved CO was then purged out of the electrolyte by bubbling N_2_ gas for 30 min. Since CO is an important intermediate product, its oxidation capability significantly influences the formic acid oxidation reaction activity. The impedance spectra were recorded between 100 kHz and 10 mHz with the amplitude (rms value) of the ac signal of 10 mV. The solutions were deaerated by bubbling ultra-high-purity N_2_ for 30 min and protected with a nitrogen atmosphere during the entire experimental procedure. All electrochemical experiments were carried out at room temperature.

### 4.6. DFT Calculation

All density functional theory (DFT) calculations were performed with Vienna Ab Initio Simulation Package (VASP) code [26, 27]. Exchange-correlation interactions were described by the Perdew-Burke-Ernzerhof functional (PBE) within the generalized gradient approximation [28], and electron-ion interactions were described by the projector-augmented plane-wave (PAW) approach [29, 30]. The cutoff energy of 400 eV was used in all computations. A vacuum layer of 35 Å was used to avoid the interactions between the neighboring images. For Pt/Sb and Pt/C models, we constructed Pt_10_ cluster on the bilayer Sb and carbon nanosheets, respectively (Sb sheets with a size of 13.14 × 13.14 Å^2^, carbon support was taken graphene configuration with a size of 12.30 × 12.30 Å^2^). The Brillouin zone was sampled with 2 × 2 × 1*k*-points for geometry calculations. The convergence threshold was conducted as 10^−4^ eV in energy and 0.05 eV/Å in force. We adopted the DFT-D3 (D stands for dispersion) method with the Grimme vdW correction to describe the weak interactions [31]. The solvent effect on adsorbates was simulated using the Poisson-Boltzmann implicit solvation model with a dielectric constant of 80 [32].

Generally, the FAOR reaction mechanism can be written in the following three paths. Path 1 is direct oxidation via OCHO^∗^. Path 2 is direct oxidation via COOH^∗^. Path 3 is indirect mechanism via COOH^∗^ which includes the dehydration step.

(1) $$\begin{array}{c} \begin{array}{cc} \text{Path }1 & \text{HCOOH}\left(\text{g}\right){+}^{*}⟶{\text{OCHO}}^{*}+{\text{H}}^{+}+{\text{e}}^{-}\\ & {\text{OCHO}}^{*}⟶{\text{CO}}_{2}\;\left(\text{g}\right)\;+\;{\text{H}}^{+}\;+\;{\text{e}}^{-}\\ \text{Path }2 & \text{HCOOH}\left(\text{g}\right){+}^{*}⟶{\text{COOH}}^{*}+{\text{H}}^{+}+{\text{e}}^{-}\\ & {\text{COOH}}^{*}⟶\;{\text{CO}}_{2}\;\left(\text{g}\right)\;+\;{\text{H}}^{+}\;+\;{\text{e}}^{-}\\ \text{Path }3 & \text{HCOOH}\left(\text{g}\right){+}^{*}⟶{\text{COOH}}^{*}+{\text{H}}^{+}+{\text{e}}^{-}\\ & {\text{COOH}}^{*}+\;{\text{H}}^{+}\;+\;{\text{e}}^{-}⟶\;{\text{CO}}^{*}\;+\;{\text{H}}_{2}\text{O }\left(\text{g}\right)\\ & {\text{H}}_{2}\text{O }+{\;}^{*}⟶\;{\text{OH}}^{*}\;+\;{\text{H}}^{+}\;+\;{\text{e}}^{-}\\ & {\text{CO}}^{*}\;+\;{\text{OH}}^{*}\;⟶\;{\text{CO}}_{2}\left(\text{g}\right)\;+\;{\text{H}}^{+}\;+\;{\text{e}}^{-} \end{array} \end{array}$$

where ∗ presents an adsorption site on the catalyst, and OCHO^∗^, COOH^∗^, CO^∗^, and CO^∗^ + OH^∗^ denote the corresponding absorbed intermediates. According to the hydrogen electrode (CHE) model, the free energy of above species can be expressed by the following equation [33]:

(2) $$\begin{array}{c} \Delta G_{\text{ads}}=\Delta E_{\text{ads}}+\Delta E_{{\text{ZPE}}^{-}}T\Delta S, \end{array}$$

where Δ*E*_ads_ is the adsorption energy change calculated from DFT, *E*_ZPE_ is the zero energy calculated from the vibrational frequencies, Δ*S* is the entropy change, and *T* is the system temperature (298.15 K, in our work). For adsorbates, all 3 N degrees of freedom are treated as harmonic vibrational motions with neglecting contributions from the slab. The calculated *E*_ZPE_ and *T*Δ*S* for each gas-phase species and adsorbates were listed in [Supplementary-material supplementary-material-1].

## Data Availability

All data needed to evaluate the conclusions in the paper are present in the paper and the Supplementary Materials. Additional data related to this paper may be requested from the authors.

## Supplementary Materials

Supplementary Materials Figure S1: (A) XRD spectra of Sb nanosheets compared with bulk Sb and Sb microcrystals. (B and C) SEM images of bulk Sb without any treatment. Inset: photograph of bulk materials. (D) SEM image of the Sb microcrystal. (E) SEM image of antimonene. (F) Atomic structure of antimonene. Figure S2: (A) AFM image of antimonene sheets. (B) The height analysis of various sizes Sb nanosheets and the mean thickness of antimonene is about 7~9 nm. We can see that 89% few layer sheets have a thickness below 9 nm and 12% of these sheets are thinner than 5 nm. Figure S3: (A) SEM image, (B) STEM-HAADF and TEM-mapping, and (C) EDX of Pt/Sb. Figure S4: (A) XRD spectra. (B) SEM image, (C) TEM, and (D) HRTEM of Pt/Ct which is prepared by the one-pot solvothermal reaction with EDA. Figure S5: the Pt 4f spectrum of Pt/C and Pt/Sb. Figure S6: shows the *k*^3^-weighted EXAFS spectra from *k* = 2.5 to 13.5 Å^−1^ of Pt/Sb (A) and Pt/C (B) (black solid lines). The best fits (red dash lines) to the spectra are also displayed in Figure S6. Figure S7: formic acid oxidation performance of the Pt/C and Pt/Sb. (A) The CV curve measured in 0.1 M HClO4 electrolyte at a scan rate of 50 mV s^−1^. (B) The CV curve measured in 0.1 M HClO4+0.1 M HCOOH electrolyte at a scan rate of 50 mV s^−1^. (C) CO stripping testing in 0.1 M HClO4 electrolyte at a scan rate of 10 mV s^−1^. (D) The chronoamperometry curves measured in 0.1 M HClO4+0.1 M HCOOH at 0.6 V (vs. RHE). Figure S8: current densities for the direct pathway (I) and the indirect pathway (II) in electrocatalytic formic acid oxidation by various catalysts. Figure S9: characterization of Pt/Sb after cycling. (A) TEM and HRTEM images. (B) EDX. (C) XRD spectra. (D) XPS survey. (E) Sb 3d spectrum. (F) Pt 4f spectrum. Figure S10: equivalent circuits for the electrooxidation of formic acid at Pt/Sb and Pt/C: (A) for normal impedance and (B) for negative impedance shown in the Nyquist plots. (C) Nyquist plots for the Pt/C catalyst in formic acid electrooxidation at different potentials (vs. RHE). (D) Nyquist plots for the Pt/Sb catalyst in formic acid electrooxidation at different potentials (vs. RHE). RS is the solution resistance, CPE (constant-phase element) and R_CT_ are the double-layer capacitance and charge-transfer resistance, respectively, and C_o_ and R_o_ represent the capacitance and resistance of the electrooxidation of adsorbed CO intermediates. Figure S11: (A) electrochemical impedance spectroscopy (EIS) of Pt/C and Pt/Sb was employed from 100 kHz to 10 mHz at the open circuit potential (before any electrochemical cycling) in 0.1 M HClO4 solution to investigate the conductivity and charge transport of Pt/Sb electrode. The open circles represent experimentally obtained data, and the solid lines are the fit of the equivalent circuit. EIS fitting results of (B) Pt/C and C) Pt/Sb (Rs: electrolyte resistance, Rp: charge-transfer resistance, and CPE: constant-phase element). Figure S12: the calculated free energy diagrams for FAOR on (A) Pt (111), (B) Pt/C, and (C) Pt/Sb. Blue, purple, white, gray, and red balls present Pt, Sb, H, C, and O atoms, respectively. Table S1: best fit values for the EXAFS analysis of Pt/Sb and Pt/C samples. Table S2: fitting parameters of the electrochemical impedance for Pt/Sb at various potentials. Table S3: fitting parameters of the electrochemical impedance for Pt/C at various potentials. Table S4: calculated zero-pint energy correction (EZPE), entropy contribution (TS), and the total free energy correction (G-Eelec) of the studied systems.
