# Supplementary material for: In Situ Exfoliation and Pt Deposition of Antimonene for Formic Acid Oxidation via a Predominant Dehydrogenation Pathway
Source: Research (Wash D C). 2020 Feb 21;2020:5487237. doi: 10.34133/2020/5487237 (PMC7054718; doi:10.34133/2020/5487237)
Supplement: Supplementary Materials — Figure S1: (A) XRD spectra of Sb nanosheets compared with bulk Sb and Sb microcrystals. (B and C) SEM images of bulk Sb without any treatment. Inset: photograph of bulk materials. (D) SEM image of the Sb microcrystal. (E) SEM image of antimonene. (F) Atomic structure of antimonene. Figure S2: (A) AFM image of antimonene sheets. (B) The height analysis of various sizes Sb nanosheets and the mean thickness of antimonene is about 7~9 nm. We can see that 89% few layer sheets have a thickness below 9 nm and 12% of these sheets are thinner than 5 nm. Figure S3: (A) SEM image, (B) STEM-HAADF and TEM-mapping, and (C) EDX of Pt/Sb. Figure S4: (A) XRD spectra. (B) SEM image, (C) TEM, and (D) HRTEM of Pt/Ct which is prepared by the one-pot solvothermal reaction with EDA. Figure S5: the Pt 4f spectrum of Pt/C and Pt/Sb. Figure S6: shows the k3-weighted EXAFS spectra from k = 2.5 to 13.5 Å−1 of Pt/Sb (A) and Pt/C (B) (black solid lines). The best fits (red dash lines) to the spectra are also displayed in Figure S6. Figure S7: formic acid oxidation performance of the Pt/C and Pt/Sb. (A) The CV curve measured in 0.1 M HClO4 electrolyte at a scan rate of 50 mV s−1. (B) The CV curve measured in 0.1 M HClO4+0.1 M HCOOH electrolyte at a scan rate of 50 mV s−1. (C) CO stripping testing in 0.1 M HClO4 electrolyte at a scan rate of 10 mV s−1. (D) The chronoamperometry curves measured in 0.1 M HClO4+0.1 M HCOOH at 0.6 V (vs. RHE). Figure S8: current densities for the direct pathway (I) and the indirect pathway (II) in electrocatalytic formic acid oxidation by various catalysts. Figure S9: characterization of Pt/Sb after cycling. (A) TEM and HRTEM images. (B) EDX. (C) XRD spectra. (D) XPS survey. (E) Sb 3d spectrum. (F) Pt 4f spectrum. Figure S10: equivalent circuits for the electrooxidation of formic acid at Pt/Sb and Pt/C: (A) for normal impedance and (B) for negative impedance shown in the Nyquist plots. (C) Nyquist plots for the Pt/C catalyst in formic acid electrooxidation at differen [file 5487237.f1.doc]

Supplementary Materials for *Research*

**In-situ Exfoliation and Pt deposition of** **Antimonene for Formic Acid Oxidation via a Predominant Dehydrogenation Pathway**

**Yiqiong Zhang,^1^**^†^ **Man Qiao,^2^**^†^ **Yucheng Huang,^3^**^†^ **Yuqin Zou,^1^,* Zhijuan Liu,^1^ Li Tao,^1^ Yafei Li,^2^,* Chung-Li Dong,^3^,* Shuangyin Wang ^1^,***

^1^ State Key Laboratory of Chemo/Bio-Sensing and Chemometrics, College of Chemistry and Chemical Engineering, Hunan University, Changsha, 410082, P. R.China

^2^ Jiangsu Collaborative Innovation Centre of Biomedical Functional Materials, School of Chemistry and Materials Science, Nanjing Normal University, Nanjing, P. R. China

^3^ Department of Physics, Tamkang University, Tamsui 25137, Taiwan

***** Correspondence should be addressed to Shuangyin Wang; [shuangyinwang@hnu.edu.cn](mailto:shuangyinwang@hnu.edu.cn) and Yafei Li; [liyafei@njnu.edu.cn](mailto:liyafei@njnu.edu.cn) and Chung-Li Dong; [cldong@mail.tku.edu.tw](mailto:cldong@mail.tku.edu.tw)

^†^ These authors contributed equally to this work


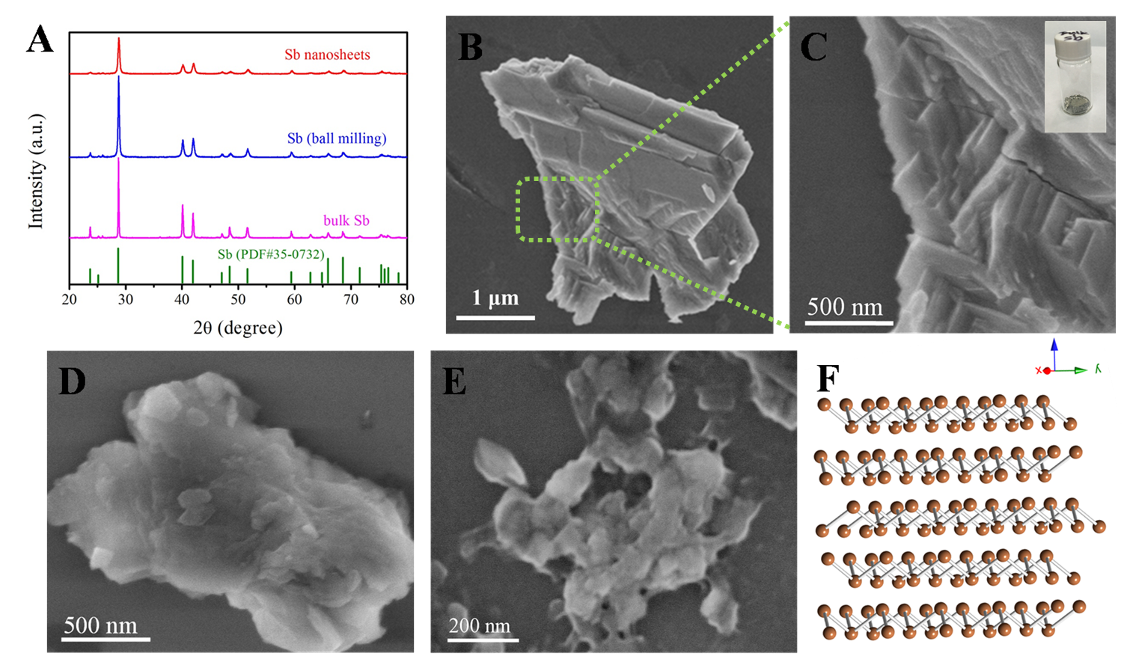


**Figure S1**. A) XRD spectra of Sb nanosheets compared with bulk Sb and Sb microcystals. B-C) SEM images of bulk Sb without any treatment. Inset: Photograph of bulk materials. D) SEM image of the Sb microcrystal. E) SEM image of antimonene. F) Atomic structure of antimonene.


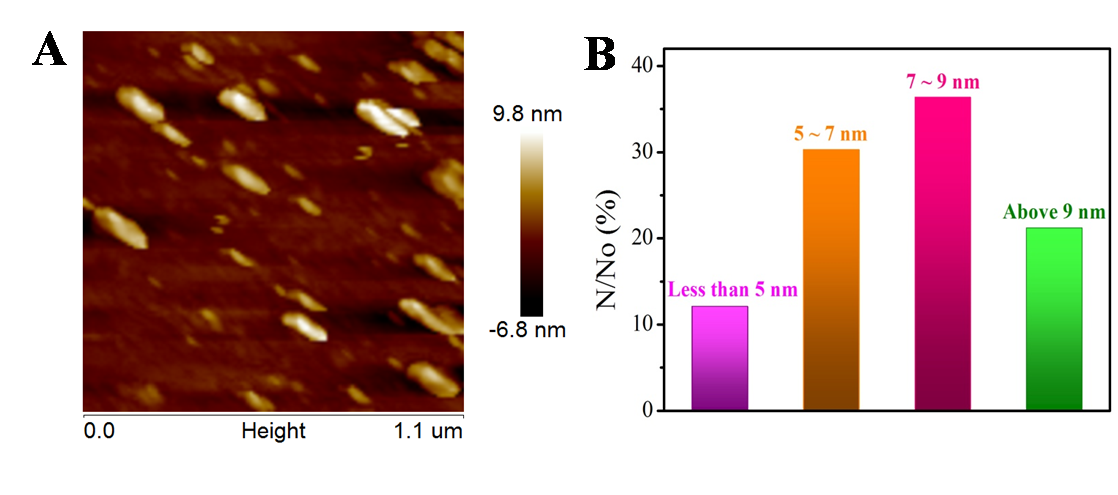


**Figure S2**. A) AFM image of antimonene sheets. B) The height analysis of various sizes Sb nanosheets and the mean thickness of antimonene is about 7~9 nm. We can see that 89 % few layer sheets have a thickness below 9 nm and 12 % of these sheets are thinner than 5 nm.


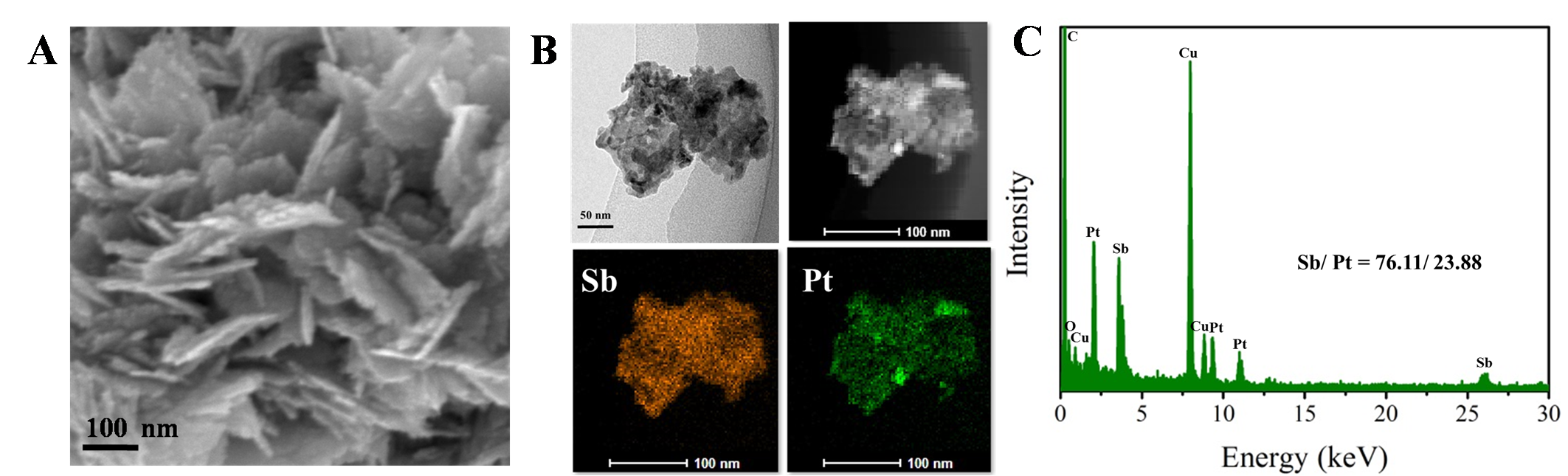


**Figure S3**. A) SEM image, B) STEM-HAADF and TEM-mapping, and C) EDX of Pt/Sb.


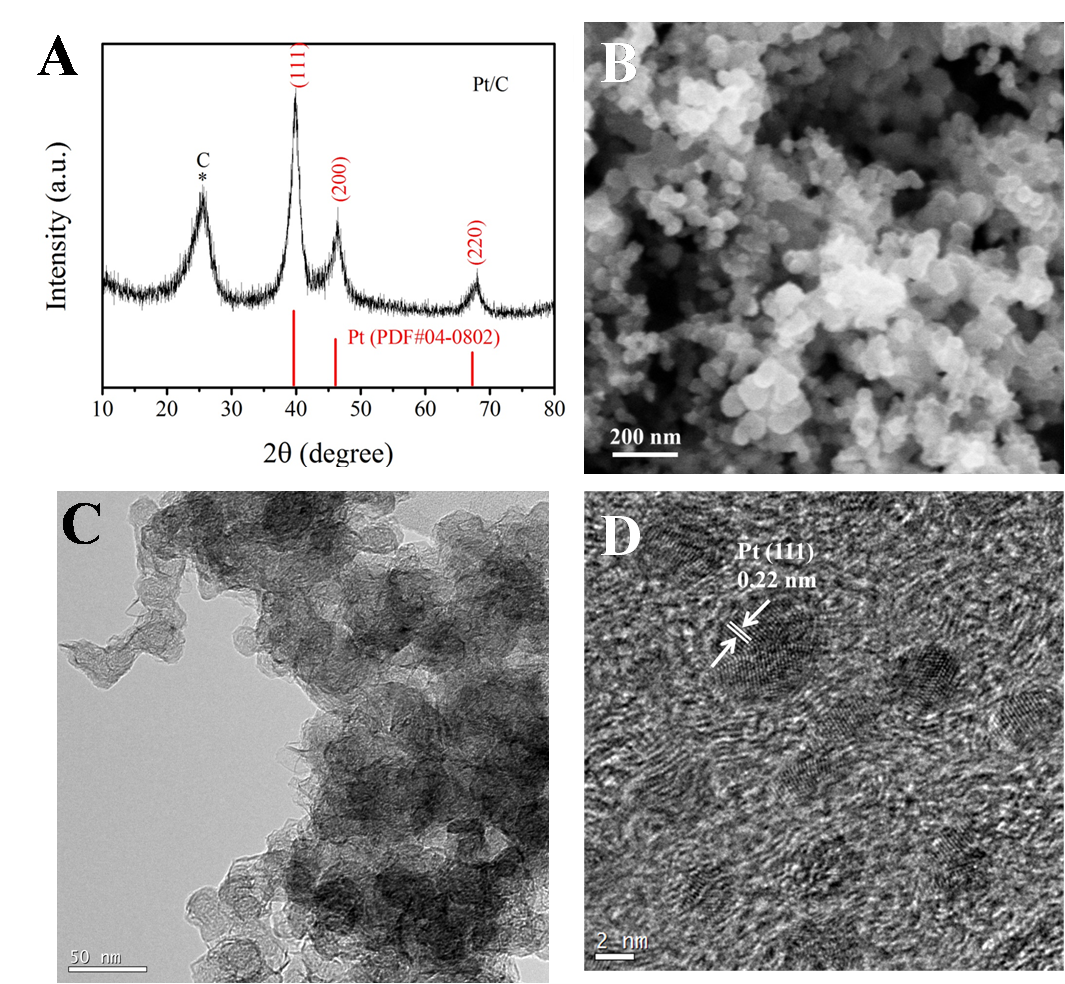


**Figure S4**. A) XRD spectra. B) SEM image, C) TEM, and D) HRTEM of Pt/Ct which is prepared by the one-pot solvothermal reaction with EDA.


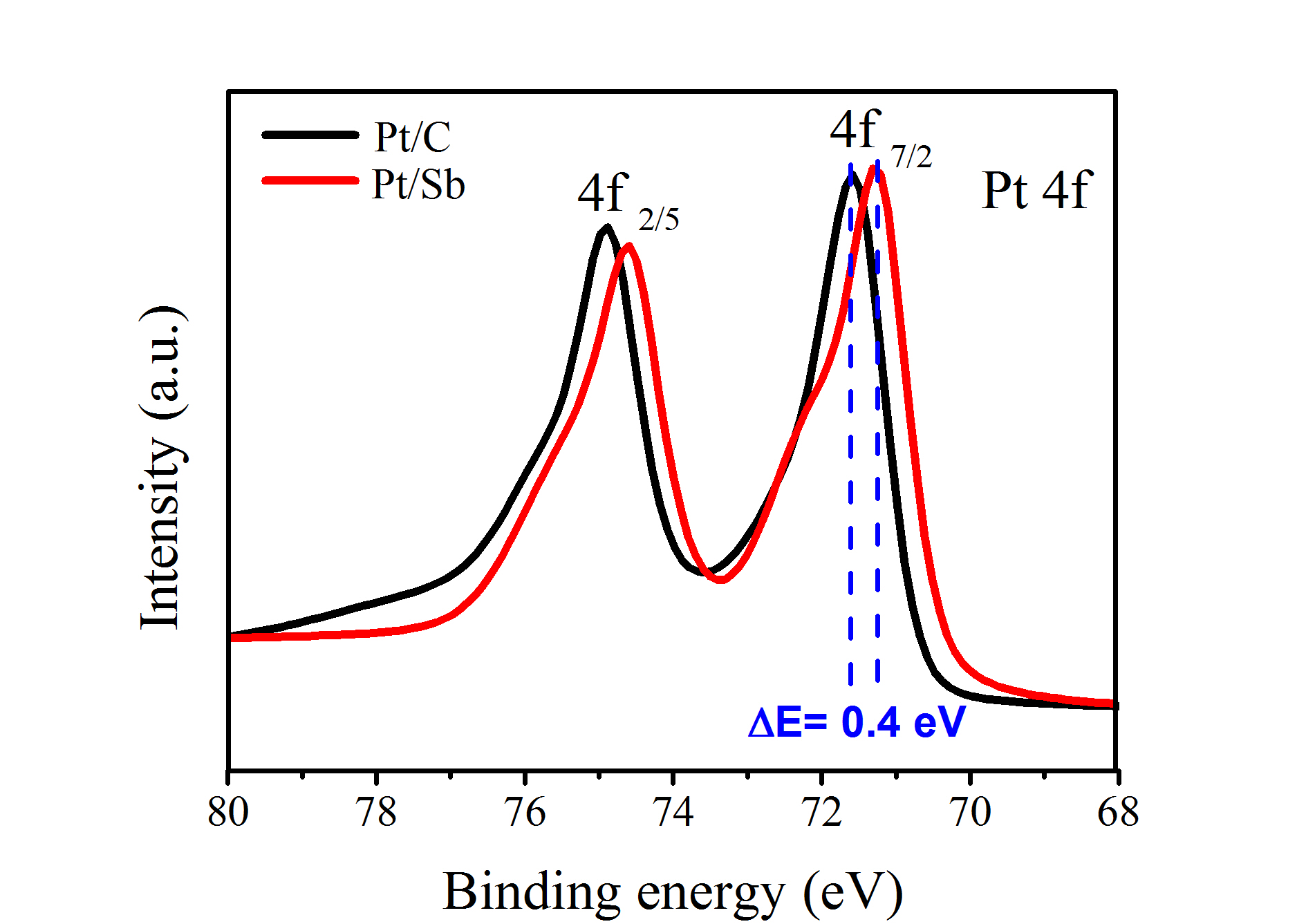


**Figure S5**. The Pt 4f spectrum of Pt/C and Pt/Sb.


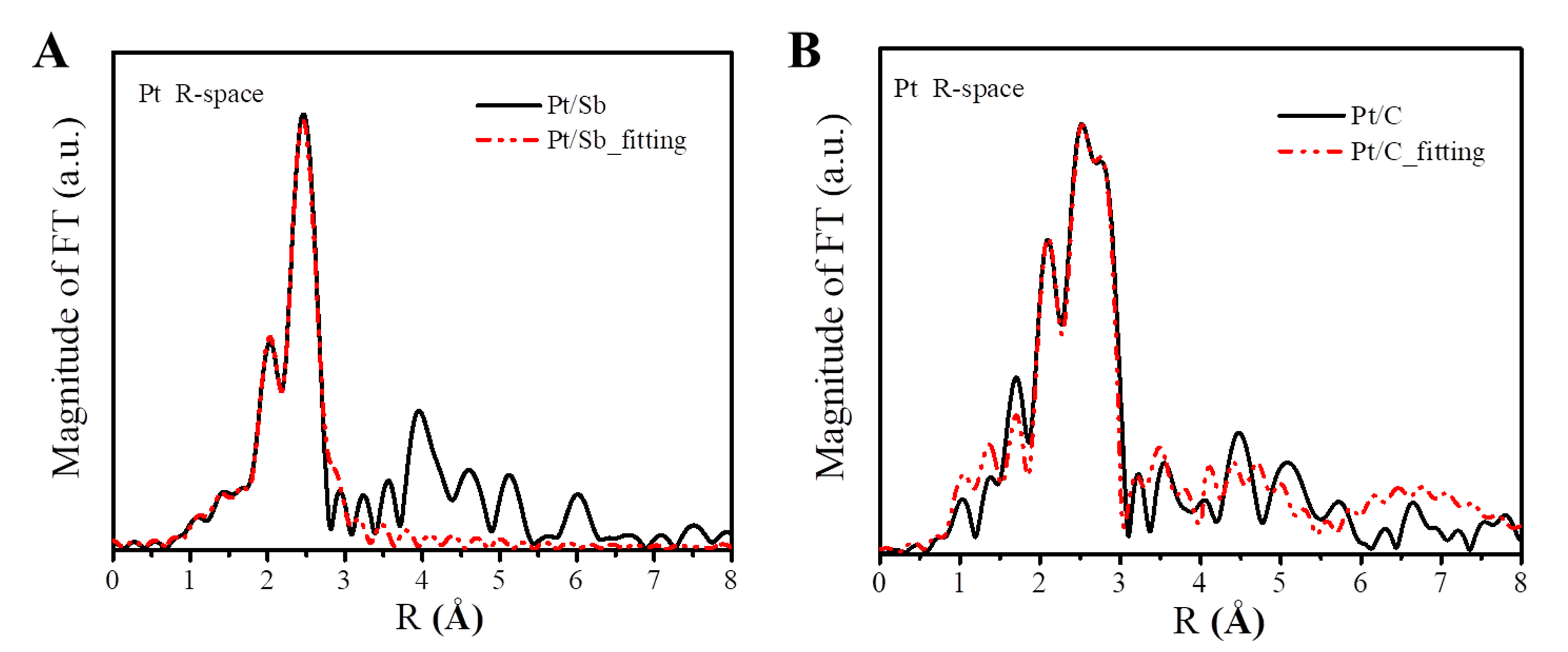


**Figure S6**. The Fourier transform k^3^-weighted extended X-ray absorption fine structure (EXAFS) spectrum of the Pt/Sb (Pt/C) shows that the first peak is attributable to Pt-Sb (Pt-C) coordination and the second peak is attributable to Pt-Pt (Pt-Pt) coordination. The quantitative curve-fittings were carried out in the k^3^-weighted EXAFS oscillation. Figure S6 shows the k^3^-weighted EXAFS spectra from k=2.5 to 13.5 Å^-1^ of Pt/Sb (A) and Pt/C (B) (black solid lines). The best fits (red dash lines) to the spectra are also displayed in Figure S6. The results of the best fits are given in **Table S1**.


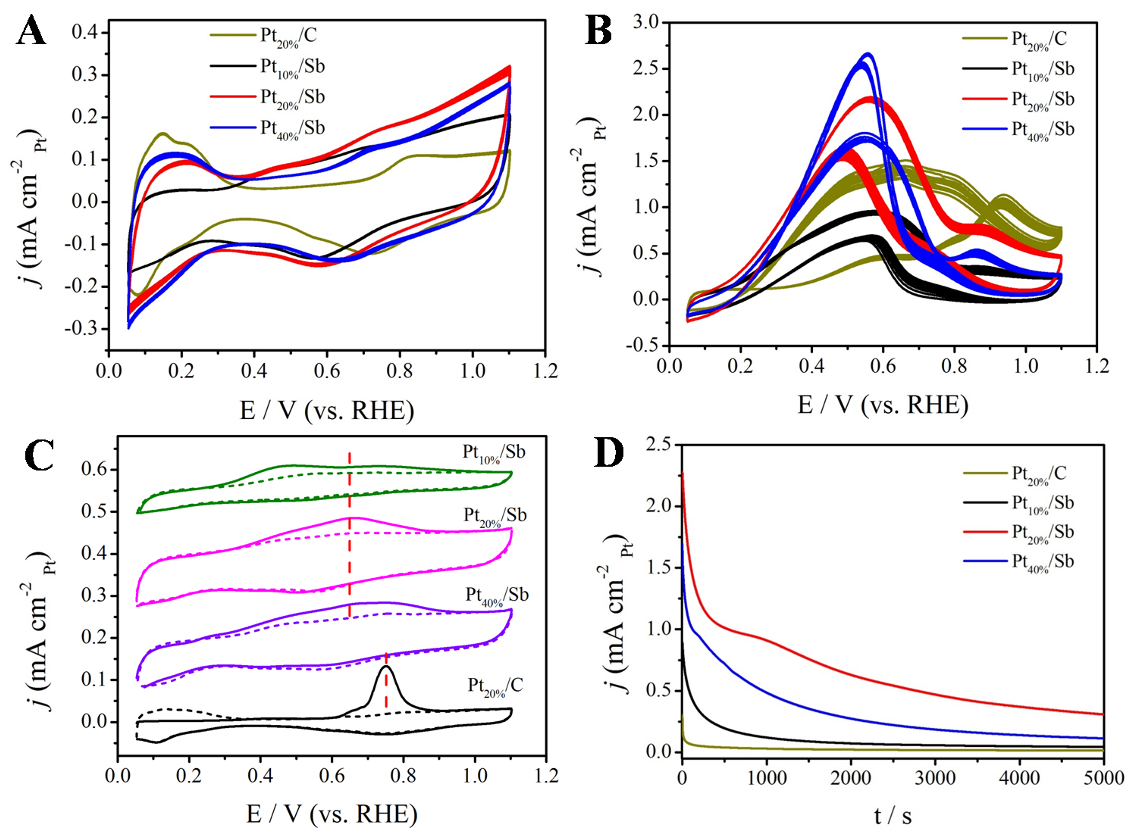


**Figure S7**. Formic acid oxidation performance of the Pt/C and Pt/Sb. A) The CV curve measured in 0.1 M HClO_4_ electrolyte at a scan rate of 50 mV s^-1^. B) The CV curve measured in 0.1 M HClO_4_+0.1 M HCOOH electrolyte at a scan rate of 50 mV s^-1^. C) CO stripping testing in 0.1 M HClO_4_ electrolyte at a scan rate of 10 mV s^-1^. D) The chronoamperometry curves measured in 0.1 M HClO_4_+0.1 M HCOOH at 0.6 V (vs. RHE).


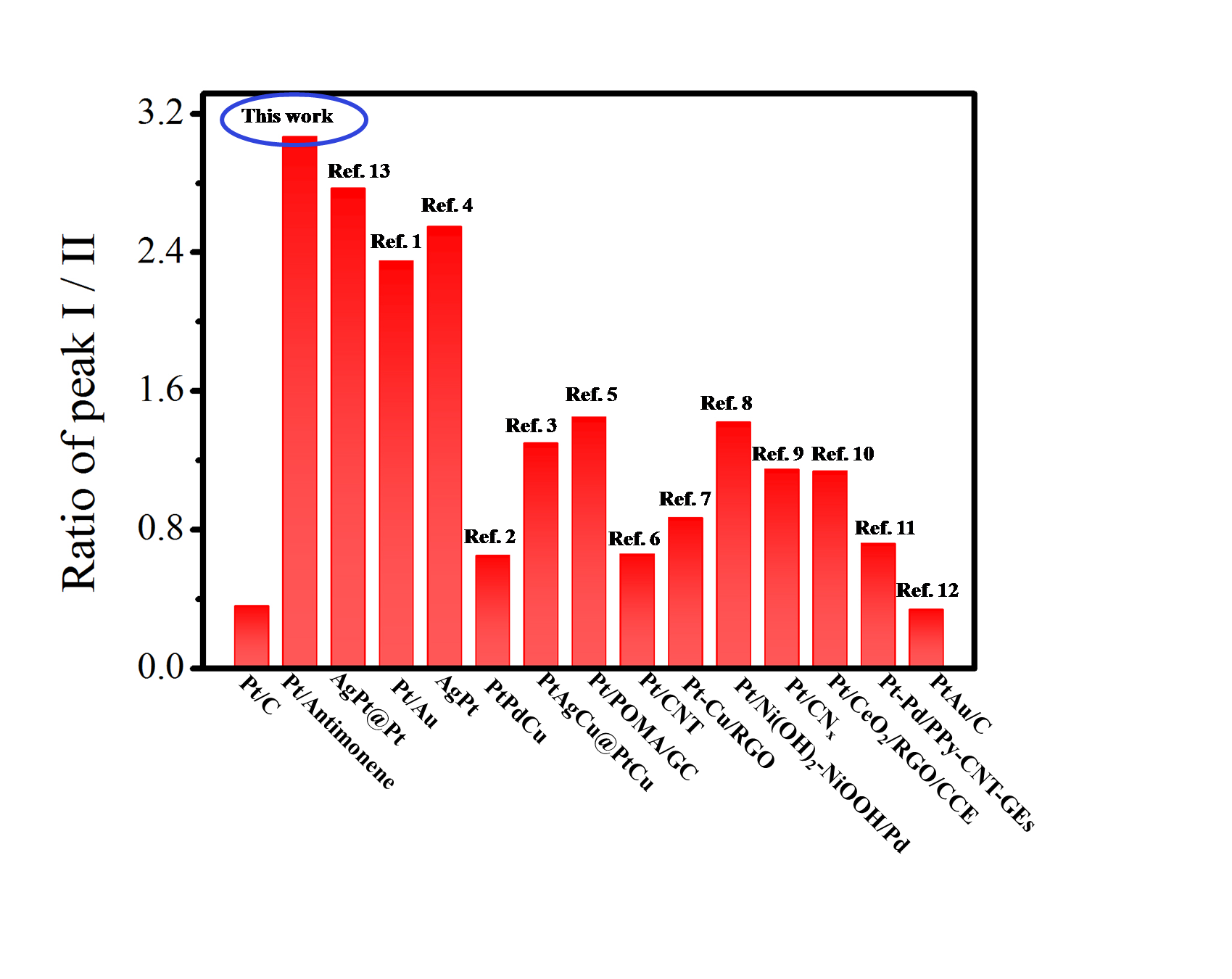


**Figure S8**. Current densities for the direct pathway (I) and indirect pathway (II) in electrocatalytic formic acid oxidation by various catalysts.^1-13^[^1^](#_ENREF_1) [^2^](#_ENREF_2) [^3^](#_ENREF_3) [^4^](#_ENREF_4) [^5^](#_ENREF_5) [^6^](#_ENREF_6) [^7^](#_ENREF_7) [^8^](#_ENREF_8) [^9^](#_ENREF_9) [^10^](#_ENREF_10) [^11^](#_ENREF_11) [^12^](#_ENREF_12) [^13^](#_ENREF_13)


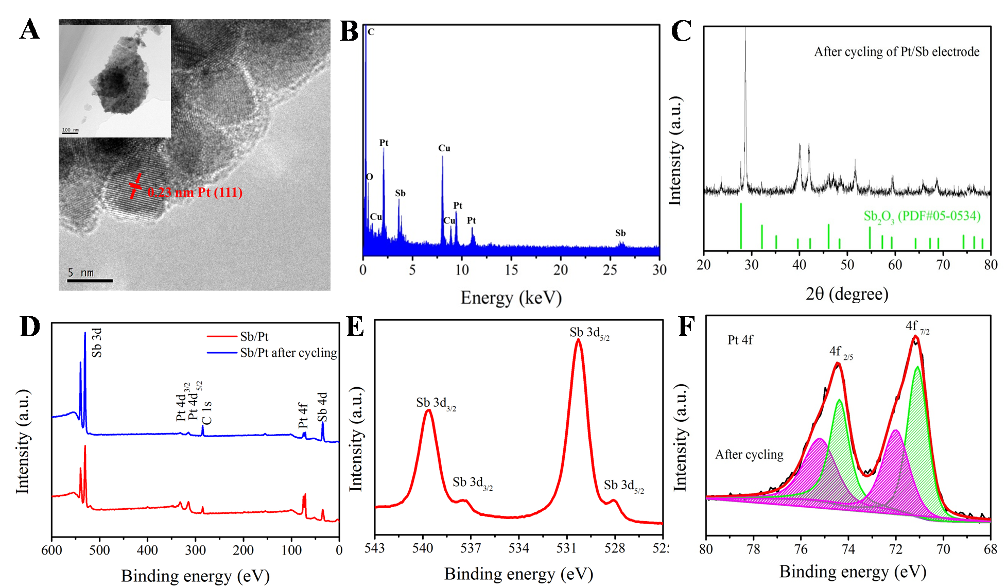


**Figure S9**. Characterization of Pt/Sb after cycling. A) TEM and HRTEM images. B) EDX. C) XRD spectra. D) XPS survey. E) Sb 3d spectrum. F) Pt 4f spectrum.


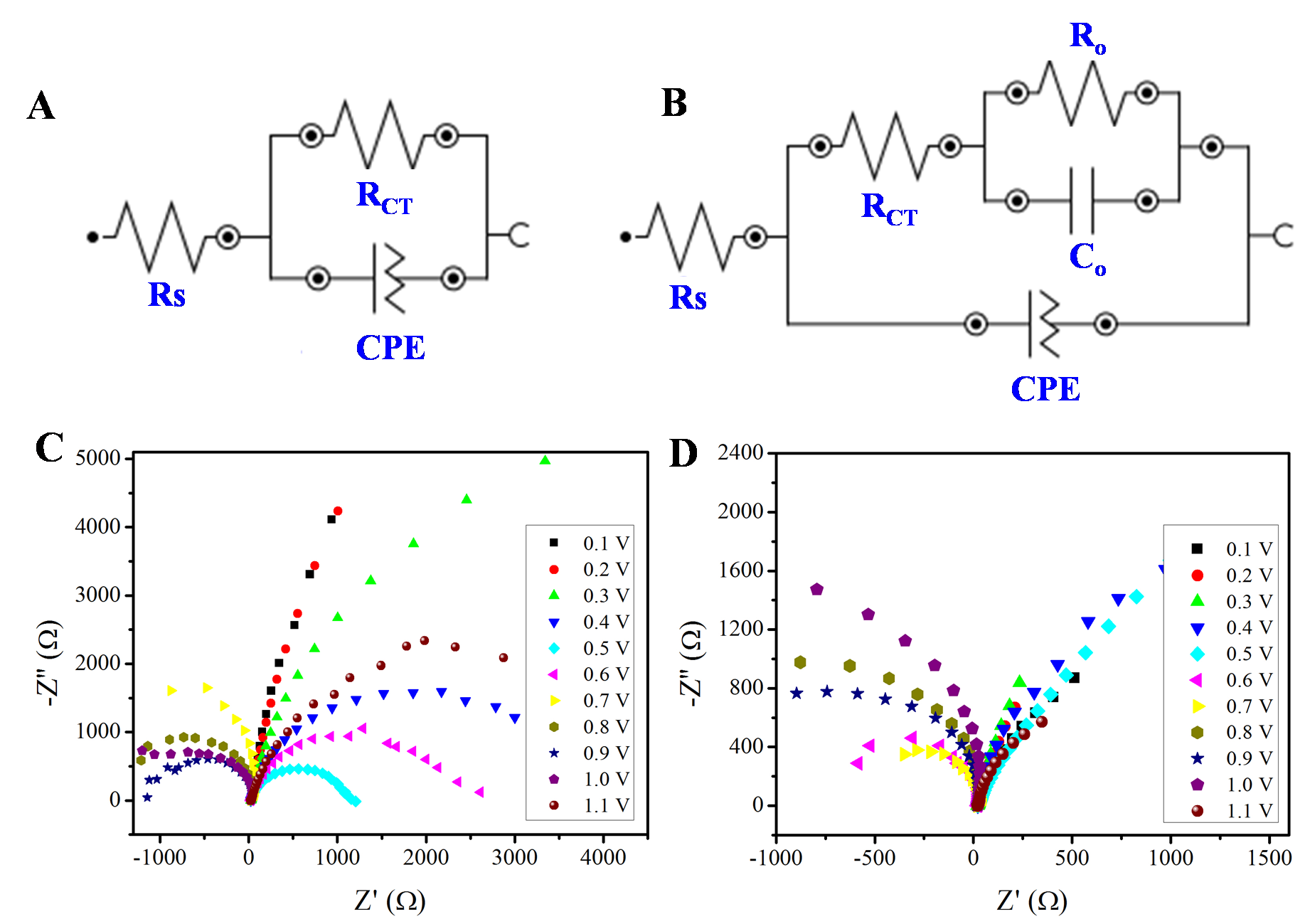


**Figure S10**. Equivalent circuits for the electro-oxidation of formic acid at Pt/Sb and Pt/C: A) for normal impedance, and B) for negative impedance shown in the Nyquist plots. C) Nyquist plots for the Pt/C catalyst in formic acid electro-oxidation at different potentials (vs.RHE). D) Nyquist plots for the Pt/Sb catalyst in formic acid electro-oxidation at different potentials (vs.RHE). R_S_ is the solution resistance, CPE (constant-phase element) and R_CT_ are the double-layer capacitance and charge-transfer resistance, respectively, and C_o_ and R_o_ represent the capacitance and resistance of the electro-oxidation of adsorbed CO intermediates.


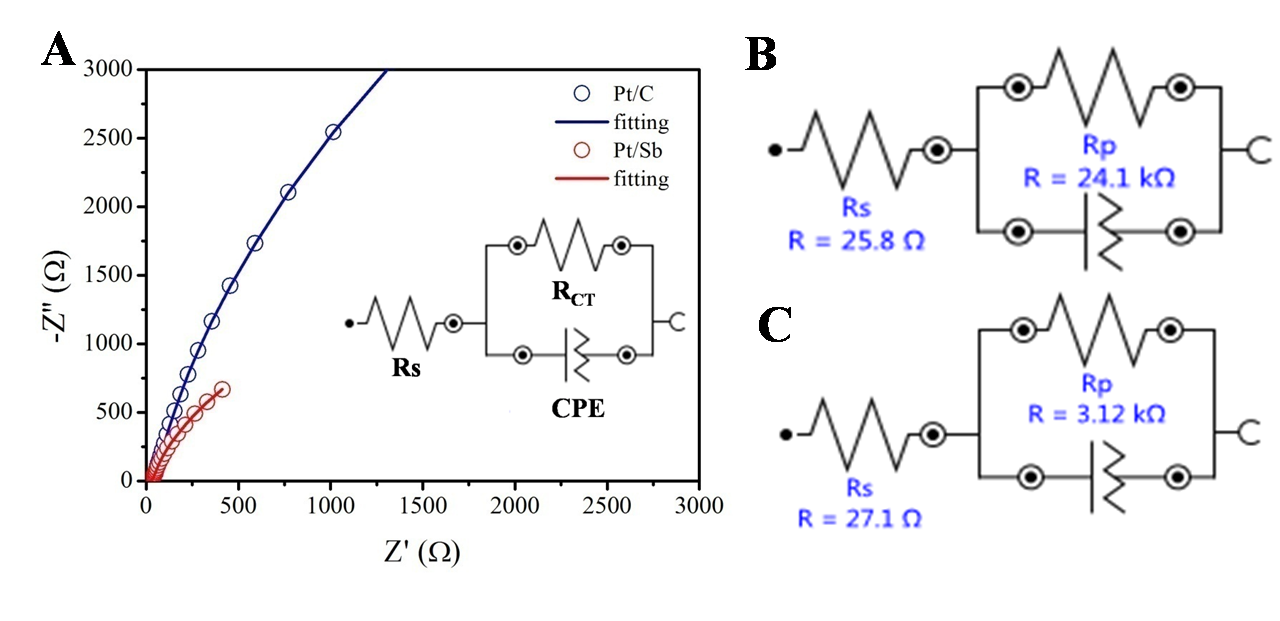


**Figure S11**. A) Electrochemical impedance spectroscopy (EIS) of Pt/C and Pt/Sb were employed from 100 kHz to 10 mHz at the open circuit potential (before any electrochemical cycling) in 0.1 M HClO_4_ solution to investigate the conductivity, and charge transport of Pt/Sb electrode. The open circles represent experimentally obtained data and the solid lines are the fit of the equivalent circuit. EIS fitting results of B) Pt/C and C) Pt/Sb. (Rs: electrolyte resistance, Rp: charge-transfer resistance, CPE: constant-phase element).


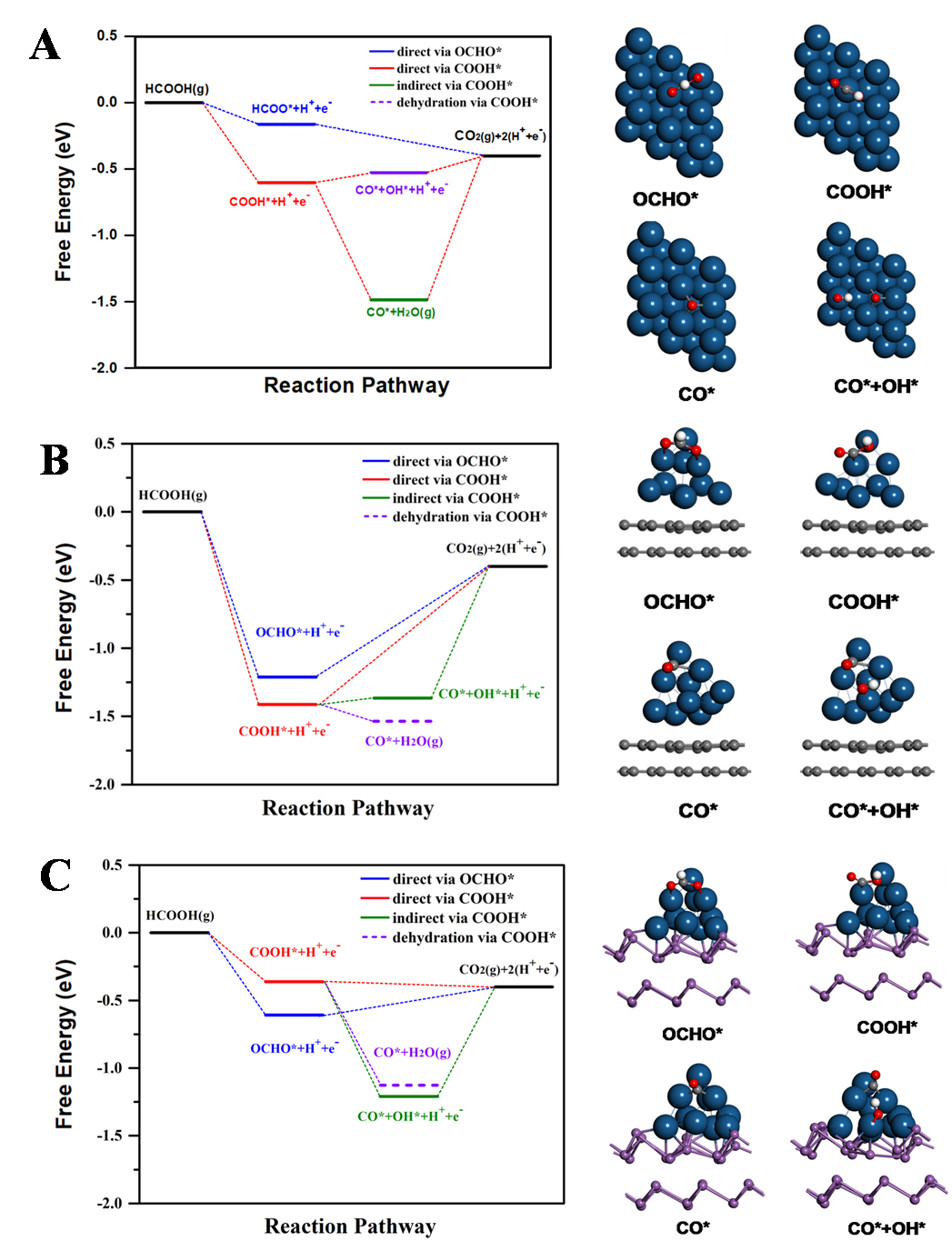


**Figure S12**. The calculated free energy diagrams for FAOR on A) Pt (111), B) Pt/C, and C) Pt/Sb. Blue, purple, white, gray and red balls present Pt, Sb, H, C and O atoms, respectively.

**Table S1.** Best fit values for the EXAFS analysis of Pt/Sb and Pt/C samples.


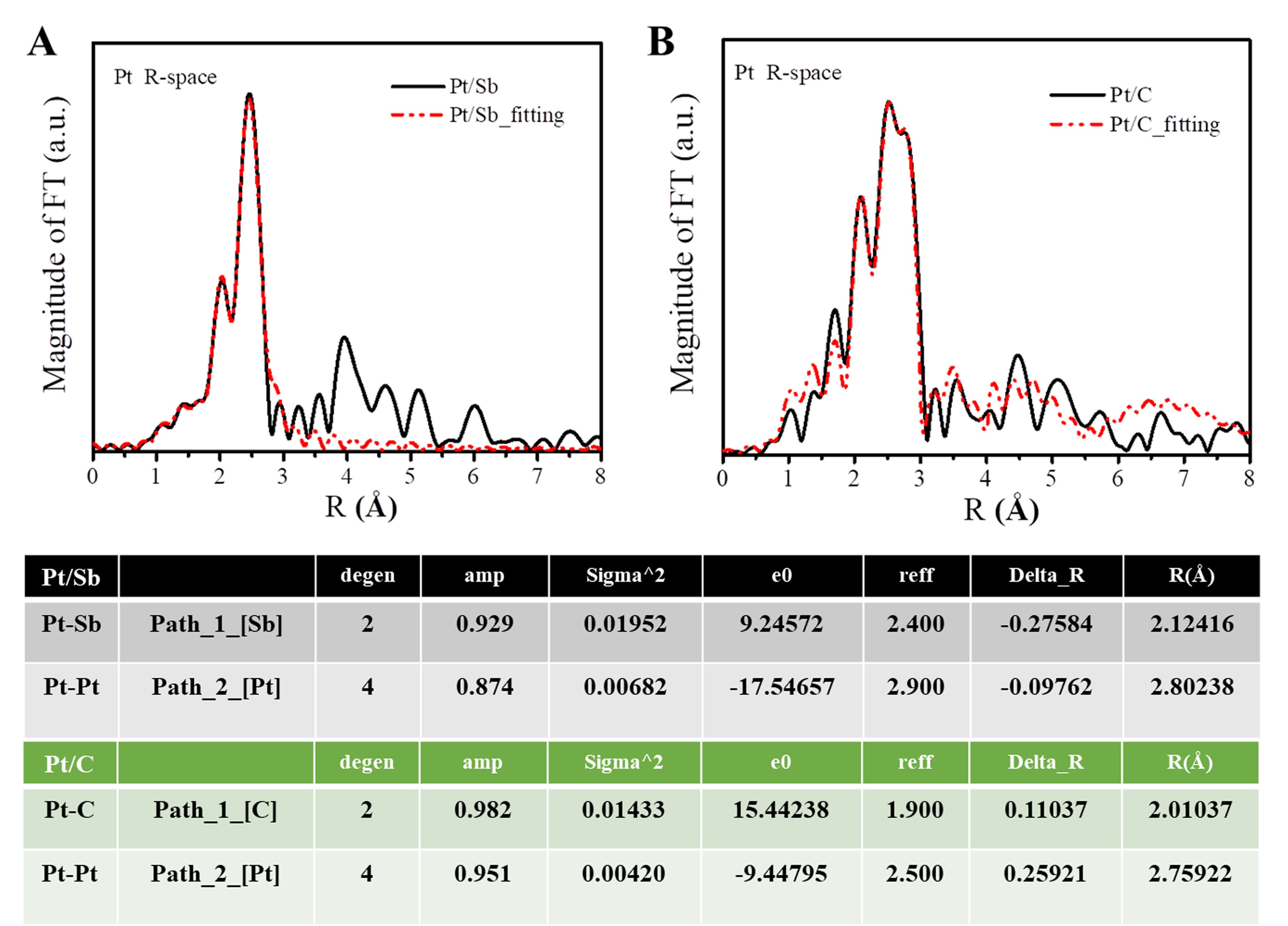


**Table S2.** Fitting parameters of the electrochemical impedance for Pt/Sb at various potentials.^a^

| **E / V**  **(vs. RHE)** | **R_s_ /Ω** | **R_CT_ / kΩ** | **CPE / μF** | **N** | **C_o_ / μF** | **R_o_ / Ω** |
| --- | --- | --- | --- | --- | --- | --- |
| **0.1** | 21.4 | 2.97 | 3.63 | 0.879 |  |  |
| **0.2** | 21.1 | 12.3 | 4.16 | 0.867 |  |  |
| **0.3** | 20.9 | 6.71 | 4.24 | 0.899 |  |  |
| **0.4** | 21.4 | 3.25 | 1.52 | 0.999 |  |  |
| **0.5** | 23.2 | 4.67 | 1.40 | 0.997 |  |  |
| **0.6** | 20.7 | -0.802 | 5.65 | 0.841 | 143 | 273 |
| **0.7** | 21.7 | -0.846 | 4.39 | 0.883 | 282 | 228 |
| **0.8** | 21.8 | -1.84 | 3.26 | 0.991 | 480 | 204 |
| **0.9** | 20.8 | -1.71 | 2.88 | 0.908 | 160 | 383 |
| **1.0** | 22.0 | -2.91 | 2.94 | 0.912 | 543 | 220 |
| **1.1** | 21.1 | 1.67 | 3.16 | 0.990 |  |  |

**Table S3.** Fitting parameters of the electrochemical impedance for Pt/C at various potentials.

| **E / V**  **(vs. RHE)** | **R_s_ /Ω** | **R_CT_ / kΩ** | **CPE / μF** | **N** | **C_o_ / μF** | **R_o_ / Ω** |
| --- | --- | --- | --- | --- | --- | --- |
| **0.1** | 29.7 | 32.9 | 0.943 | 0.999 |  |  |
| **0.2** | 36.2 | 38.9 | 0.950 | 0.998 |  |  |
| **0.3** | 21.2 | 15.2 | 2.39 | 0.998 |  |  |
| **0.4** | 30.0 | 3.68 | 2.10 | 0.996 |  |  |
| **0.5** | 26.0 | 1.06 | 2.22 | 0.998 |  |  |
| **0.6** | 35.6 | 2.21 | 2.77 | 0.999 |  |  |
| **0.7** | 23.2 | -2.96 | 3.21 | 0.832 | 51.2 | 376 |
| **0.8** | 23.1 | -1.87 | 2.96 | 0.842 | 12.5 | 412 |
| **0.9** | 23.3 | -1.30 | 3.19 | 0.836 | 20.5 | 273 |
| **1.0** | 23.8 | -1.84 | 4.03 | 0.828 | 12.7 | 786 |
| **1.1** | 27.0 | 1.79 | 3.36 | 0.998 |  |  |

^a^ Experimental data were measured in 0.1 M HCOOH + 0.1 M HClO_4_ and fitted by using the equivalent circuits shown in Figure S10.

**Table S4**. Calculated zero-pint energy correction (*E_ZPE_*), entropy contribution (*TS*), and the total free energy correction (*G- E_elec_*) of the studied systems.

| **Species** | ***E_ZPE_*** | ***∫C_p_dT* (eV)** | ***-TS* (eV)** | ***G- E_elec_*(eV)** |
| --- | --- | --- | --- | --- |
| **H_2_** | 0.27 | 0.09 | -0.42 | -0.06 |
| **CO_2_** | 0.31 | 0.12 | -0.68 | -0.25 |
| **CO** | 0.13 | 0.09 | -0.61 | -0.39 |
| **H_2_O** | 0.57 | 0.10 | -0.69 | -0.02 |
| **HCOOH** | 0.89 | 0.09 | -0.99 | -0.01 |
| **COOH* on Pt/Sb** | 0.60 | 0.09 | -0.17 | 0.52 |
| **OCHO* on Pt/Sb** | 0.63 | 0.09 | -0.18 | 0.54 |
| **CO* on Pt/Sb** | 0.20 | 0.06 | -0.13 | 0.13 |
| **CO*+ OH* on Pt/Sb** | 0.56 | 0.10 | -0.17 | 0.49 |
| **COOH* on Pt/C** | 0.63 | 0.10 | -0.23 | 0.50 |
| **OCHO* on Pt/C** | 0.52 | 0.11 | -0.22 | 0.41 |
| **CO* on Pt/C** | 0.21 | 0.04 | -0.06 | 0.19 |
| **CO*+ OH* on Pt/C** | 0.56 | 0.10 | -0.19 | 0.47 |

**References**

[1] I.-S. Park, K.-S. Lee, J.-H. Choi, H.-Y. Park and Y.-E. Sung, Surface structure of Pt-modified Au nanoparticles and electrocatalytic activity in formic acid electro-oxidation. *J. Phys. Chem. C*, **2007**, *111*, 19126-19133.

[2] W. Ye, S. Chen, M. Ye, C. Ren, J. Ma, R. Long, C. Wang, J. Yang, L. Song and Y. Xiong, Pt_4_PdCu_0.4_ alloy nanoframes as highly efficient and robust bifunctional electrocatalysts for oxygen reduction reaction and formic acid oxidation. *Nano Energy*, **2017**, *39*, 532-538.

[3] G.-T. Fu, B.-Y. Xia, R.-G. Ma, Y. Chen, Y.-W. Tang and J.-M. Lee, Trimetallic PtAgCu@PtCu core@shell concave nanooctahedrons with enhanced activity for formic acid oxidation reaction. *Nano Energy*, **2015**, *12*, 824-832.

[4] X. Jiang, G. Fu, X. Wu, Y. Liu, M. Zhang, D. Sun, L. Xu and Y. Tang, Ultrathin AgPt alloy nanowires as a high-performance electrocatalyst for formic acid oxidation. *Nano Res.*, **2018**, *11*, 499-510.

[5] F. Ren, W. Zhou, Y. Du, P. Yang, C. Wang and J. Xu, High efficient electrocatalytic oxidation of formic acid at Pt dispersed on porous poly(o-methoxyaniline). *Int. J. Hydrogen Energy*, **2011**, *36*, 6414-6421.

[6] C.-T. Hsieh, J.-L. Gu, D.-Y. Tzou, Y.-C. Chu and Y.-C. Chen, Microwave deposition of Pt catalysts on carbon nanotubes with different oxidation levels for formic acid oxidation. *Int. J. Hydrogen Energy*, **2013**, *38*, 10345-10353.

[7] F. Li, Y. Guo, Y. Liu, H. Qiu, X. Sun, W. Wang, Y. Liu and J. Gao, Fabrication of Pt-Cu/RGO hybrids and their electrochemical performance for the oxidation of methanol and formic acid in acid media. *Carbon*, **2013**, *64*, 11-19.

[8] H. Xu, L.-X. Ding, J.-X. Feng and G.-R. Li, Pt/Ni(OH)_2_-NiOOH/Pd multi-walled hollow nanorod arrays as superior electrocatalysts for formic acid electrooxidation. *Chem. Sci.*, **2015**, *6*, 6991-6998.

[9] M. Sadhukhan, M. K. Kundu, T. Bhowmik and S. Barman, Highly dispersed platinum nanoparticles on graphitic carbon nitride: a highly active and durable electrocatalyst for oxidation of methanol, formic acid and formaldehyde. *Int. J. Hydrogen Energy*, **2017**, *42*, 9371-9383.

[10] B. Habibi and N. Delnavaz, Pt-CeO_2_/reduced graphene oxide nanocomposite for the electrooxidation of formic acid and formaldehyde. *RSC Adv.*, **2015**, *5*, 73639-73650.

[11] V. Selvaraj, M. Alagar and K. S. Kumar, Synthesis and characterization of metal nanoparticles-decorated PPY-CNT composite and their electrocatalytic oxidation of formic acid and formaldehyde for fuel cell applications. *Appl. Catal. B: Environ.*, **2007**, *75*, 129-138.

[12] N. Kristian, Y. Yan and X. Wang, Highly efficient submonolayer Pt-decorated Au nano-catalysts for formic acid oxidation. *Chem. Commun.*, **2008**, 353-355.

[13] X. Jiang, X. Yan, W. Ren, Y. Jia, J. Chen, D. Sun, L. Xu and Y. Tang, Porous AgPt@Pt nanooctahedra as an efficient catalyst toward formic acid oxidation with predominant dehydrogenation pathway. *ACS Appl. Mater. Inter.*, **2016**, *8*, 31076-31082.
